# *Agrobacterium tumefaciens*-Mediated Nuclear Transformation of a Biotechnologically Important Microalga—*Euglena gracilis*

**DOI:** 10.3390/ijms22126299

**Published:** 2021-06-11

**Authors:** Ina Becker, Binod Prasad, Maria Ntefidou, Viktor Daiker, Peter Richter, Michael Lebert

**Affiliations:** Department of Cell Biology, University Erlangen-Nuremberg, 91058 Erlangen-Nuremberg, Germany; ina_becker89@web.de (I.B.); maria.ntefidou@fau.de (M.N.); viktor.daiker@fau.de (V.D.); peter.richter@fau.de (P.R.); michael.lebert@fau.de (M.L.)

**Keywords:** *Euglena gracilis*, *Agrobacterium tumefaciens*, transformation, genetic engineering, gene silencing

## Abstract

*Euglena gracilis* (*E. gracilis*) is an attractive organism due to its evolutionary history and substantial potential to produce biochemicals of commercial importance. This study describes the establishment of an optimized protocol for the genetic transformation of *E. gracilis* mediated by *Agrobacterium* (*A. tumefaciens*). *E. gracilis* was found to be highly sensitive to hygromycin and zeocin, thus offering a set of resistance marker genes for the selection of transformants. *A. tumefaciens*-mediated transformation (ATMT) yielded hygromycin-resistant cells. However, hygromycin-resistant cells hosting the *gus* gene (encoding β-glucuronidase (GUS)) were found to be GUS-negative, indicating that the *gus* gene had explicitly been silenced. To circumvent transgene silencing, GUS was expressed from the nuclear genome as transcriptional fusions with the hygromycin resistance gene (*hptII*) (encoding hygromycin phosphotransferase II) with the foot and mouth disease virus (FMDV)-derived 2A self-cleaving sequence placed between the coding sequences. ATMT of *Euglena* with the *hptII-2A–gus* gene yielded hygromycin-resistant, GUS-positive cells. The transformation was verified by PCR amplification of the T-DNA region genes, determination of GUS activity, and indirect immunofluorescence assays. Cocultivation factors optimization revealed that a higher number of transformants was obtained when *A. tumefaciens* LBA4404 (A_600_ = 1.0) and *E. gracilis* (A_750_ = 2.0) cultures were cocultured for 48 h at 19 °C in an organic medium (pH 6.5) containing 50 µM acetosyringone. Transformation efficiency of 8.26 ± 4.9% was achieved under the optimized cocultivation parameters. The molecular toolkits and method presented here can be used to bioengineer *E. gracilis* for producing high-value products and fundamental studies.

## 1. Introduction

The unicellular freshwater flagellate *Euglena gracilis* is a mixotroph (facultative photoautotroph) and has multiple modes of movement, including flagellum-based swimming and euglenoid locomotion [1]. *E. gracilis* uses several external stimuli to orient in their environment. It exhibits marked phototaxis and gravitaxis: both responses include signaling and cellular mechanisms [2,3,4,5,6]. *E. gracilis* has been acknowledged as a model organism for gravitational research. Its behaviors (phototaxis and gravitaxis mechanisms) have been extensively investigated in various space experiments [7,8]. However, less is known about the complex and divergent signaling pathways in *Euglena*, an understanding of which will provide an unprecedented view of its ability to respond to light and gravity and different environmental challenges.

*E. gracilis* is an important organism due to its evolutionary history, genetic and cellular diversity, complex biology, and considerable potential to produce a wide array of secondary metabolites for many different biotechnological purposes [9,10,11,12,13]. It has been broadly studied to produce vitamins, essential amino acids, polyunsaturated fatty acids, and the glycopolymer β-1,3-glucan, paramylon [13,14]. The glycopolymer is reported to possess immune-stimulatory properties [15], anti-HIV activity [16,17], anti-influenza virus effect [18], anti-atopic dermatitis effect [19], hepatoprotective effect [20], anticancer properties [21], and antimicrobial activity [22]. Thus, *Euglena* offers extensive opportunities for metabolic engineering to produce high-value-added biomolecules. In recent years, there has been an increasing interest in utilizing *Euglena* as a biofuel producer due to its ability to manufacture waxes suitable for combustion [9,23]. Furthermore, *E. gracilis* has incited its application as a bioremediation agent [10,24,25,26] because of its ability to adapt and grow despite various environmental challenges, including low pH and high metal ion concentrations.

Despite the well-established potential for biotechnology applications, the absence of a complete genome sequence has dramatically hindered efforts to develop genetic tools and improve *E. gracilis* for expanding its biotechnological potential. Only 20% of the genome assembly or coding sequence ratio has been estimated, supporting the genome’s initial features [27,28]. Efficient molecular toolkits and transformation methods are limited for *E. gracilis* bioengineering compared to other microorganisms of commercial importance [26,29]. Several groups have reported the introduction of double-stranded RNA into *Euglena* by electroporation and degradation of specific mRNA by the RNAi approach [2,30,31,32]. Very few reports are available on genome engineering of *E. gracilis* by biolistic, electroporation, and *Agrobacterium*-mediated transformation approaches [29,33]. Very recently, a successful attempt to develop transgene-free *E. gracilis* using CRISPR/Cas9 ribonucleoproteins has been reported [34]. The CRISPR/Cas9 method is efficient in producing knockouts; however, the approach has disadvantages to perform knockin of larger inserts. Establishing an efficient transformation method and advanced genetic toolkits for metabolic engineering of *E. gracilis* would increase the potential value of this alga in biotechnological applications, and it could serve as a molecular model for freshwater Euglenophyceae.

In this study, we report an efficient *A. tumefaciens*-mediated transformation (ATMT) approach and molecular toolkits for the genetic engineering of *E. gracilis* KLEBS strain Z. *A. tumefaciens* has been efficiently used to transform its natural host plants and other organisms, including microalgae from different lineages [29,35,36,37,38,39,40,41,42,43,44,45]. Genetic transformation via *A. tumefaciens* offers several advantages, including the transfer of large DNA fragments, ease of implementation, low level of rearrangement, and the lower tendency of transgene silencing due to gene integration into transcriptionally active regions and a low copy level of the transgene [43,46,47]. Initially, we carried out an extensive analysis of the sensitivity of *E. gracilis* KLEBS strain Z to a range of antibiotics to select a resistance marker gene to select transformants efficiently. Different gene cassettes of marker and reporter genes were constructed in the T-DNA region of binary vectors and implemented strategies to overcome the challenge of low transgene expression from the nuclear genome. Furthermore, the cocultivation factors that influence the efficiency of genetic transformation were optimized. The optimized ATMT strategy and the genetic toolkits presented in this study to obtain transgenic *E. gracilis* would be helpful for further genetic engineering of this promising microalga for industrial applications.

## 2. Results

### 2.1. Sensitivity of E. gracilis and A. tumefaciens to Different Antibiotics

**Figure 1:**
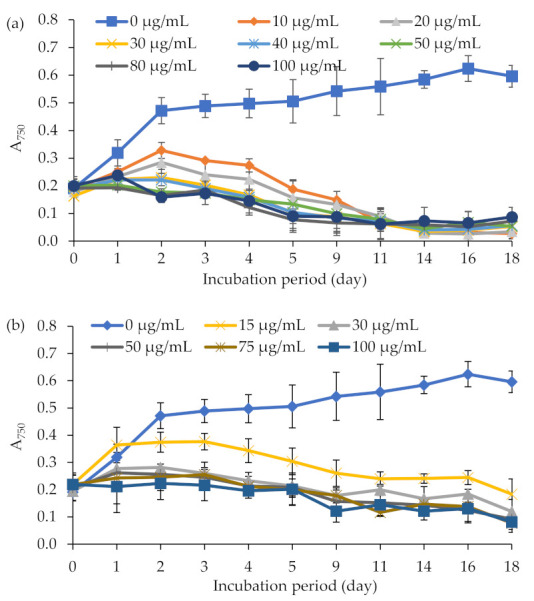
Growth inhibitory effects of the antibiotics (**a**) hygromycin (0, 10, 30, 40, 50, 80, and 100 µg mL^−1^) and (**b**) zeocin (0, 15, 30, 50, 75, and 100 µg mL^−1^) on *E. gracilis*. The growth of *E. gracilis* was assayed by measuring culture absorbance at 750 nm. Each value indicates the mean ± standard deviations of three replicates.

Knowledge about the sensitivity of a given strain to antibiotics is imperative to select a resistance marker gene for efficient screening and selection of transformants. Thus, initially, we analyzed the sensitivity of *E. gracilis* to a range of antibiotics, including hygromycin, zeocin, and kanamycin. Growth rates of *E. gracilis* at various concentrations of antibiotics (the Materials and Methods section) were assessed by multiple measurements of absorbance at 750 nm. *E. gracilis* was found sensitive to the antibiotics hygromycin and zeocin. These antibiotics caused a 55–70% reduction in growth at the concentration of 10 μg mL^−1^ (for hygromycin) and 15 μg mL^−1^ (for zeocin) within seven days (Figure 1a,b). Concentrations ≥ 30 µg mL^−1^ (for both hygromycin and zeocin) completely inhibited *E. gracilis* growth. The culture was insensitive to the tested concentrations (0, 20, 50, 100, or 200 µg mL^−1^) of the antibiotic kanamycin (Figure S1a). The cells exposed to concentrations up to 200 μg mL^−1^ of kanamycin grew identical to the control. The toxicity of different concentrations of ticarcillin/clavulanate (0, 250, or 500 µg mL^−1^) and cefotaxime (0, 250, or 500 µg mL^−1^) on *E. gracilis* and *Agrobacterium* strains LBA4404, AGL-1, and C58C1 were also tested to determine the effective concentration required to eliminate *Agrobacterium* cells after cocultivation and transformation. The antibiotics ticarcillin/clavulanate or cefotaxime did not affect *E. gracilis* growth as observed by the identical growth behavior (A_750_) in the medium with and without antibiotics (Figure S1b,c). However, concentrations ≥ 500 μg mL^−1^ of these antibiotics completely inhibited the growth of the *Agrobacterium* strains LBA4404, AGL-1, and C58C1 within 24 h (Figure S2).

We also assessed the susceptibility of *E. gracilis* to the selective agents on agar plates to affirm further the lethal concentration of antibiotics appropriate for selecting transformants on plates. *E. gracilis* (1.0 × 10^5^ cells mL^−1^) was inoculated on agar plates supplemented with varying concentrations of the antibiotics, and cell viability was monitored over 3–4 weeks. However, due to the euglenoid movement of *E. gracilis* cells, it was challenging to obtain isolated colonies and reproducible data on the agar plate assay. Several attempts with increasing agar concentrations or diluting the cell concentrations to get reproducible data on single colonies were unsuccessful. Therefore, the concentration at which no colonies grew was considered to determine the lethal concentration of the antibiotics on plates. Screening on agar plates followed the same trend as in the liquid organic medium (data not shown). No colonies were observed on agar plates supplemented with ≥30 µg mL^−1^ of the antibiotics hygromycin or zeocin. In contrast, the tested concentrations of the antibiotics kanamycin, ticarcillin/clavulanate, and cefotaxime did not inhibit the growth of *E. gracilis* on agar plates as observed in the broth assay.

### 2.2. Transformation of E. gracilis via A. tumefaciens

**Figure 2:**
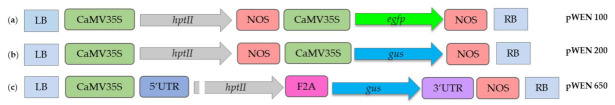
Structure of T-DNA regions of different vector systems. (**a**) The T-DNA fragment of plasmid pWEN 100 harbors the hygromycin-resistant marker gene (*hptII*) and the reporter gene (*egfp*) controlled by CaMV35S promoters. (**b**) The T-DNA region of plasmid pWEN 200 harbors the *hptII* gene and the reporter gene *gus* controlled by CaMV35S promoters. (**c**) The T-DNA region of plasmid pWEN 650 harbors the *hptII-2A-gus* open reading frame (ORF) controlled by the CaMV35S promoter and 5′UTR of the light-harvesting chlorophyll-binding protein of photosystem II (LHCPII) of *E. gracilis*. A copy of the first intron of LHCPII is placed in the *hptII* gene keeping the amino acid sequence unchanged. LB: Left border of T-DNA, RB: right border of T-DNA, CaMV35S: cauliflower mosaic virus 35S promoter, *hptII*: hygromycin phosphotransferase II, NOS: nopaline synthase terminator, *egfp*: enhanced green fluorescent protein, *gus*: β-glucuronidase, F2A: foot and mouth disease virus-derived (FMDV) 2A.

Initial transformation experiments were performed with the vector systems pWEN 100 and 200. These vectors harbor the hygromycin-resistant marker gene (*hptII*) and the reporter genes (*egfp* (encoding enhanced green fluorescent protein) in pWEN 100 or *gus* (encoding β-glucuronidase (GUS)) in pWEN 200) under the control of the CaMV35S promoter (Figure 2a,b, Figure S3). The assembly of the gene cassettes into the binary vector was done as described in the Materials and Methods section. The integrity of constructs was verified by subjecting them to various restriction endonucleases (data not shown). For the ATMT, freshly grown cells of *E. gracilis* (A_600_ = 1.0) were cocultivated with *A. tumefaciens* (A_750_ = 2.0) in an organic medium at 21 °C. After 48-h cocultivation, the cells were harvested and spread inoculated on organic medium plates containing hygromycin (50 µg mL^−1^) and ticarcillin/clavulanate (500 µg mL^−1^) and incubated at 21 °C under continuous light at 22 W m^−2^ until the colonies were observed.

**Figure 3:**
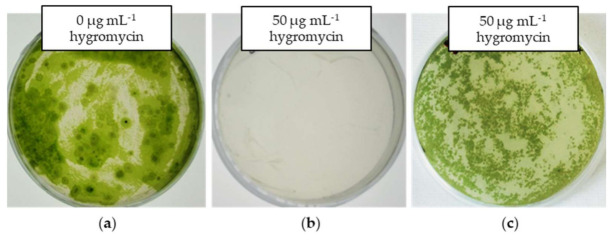
*Agrobacterium*-mediated transformed *E. gracilis* cells. (**a**) Wildtype *E. gracilis* cells on a hygromycin-free organic medium. (**b**) *E. gracilis* cells cocultivated with *Agrobacterium* cells harboring a plasmid without the resistance/reporter gene cassette and plated on an organic medium containing 50 µg mL^−1^ hygromycin. No colonies of *E. gracilis* were observed. (**c**) *E. gracilis* cells transformed with *Agrobacterium* harboring a plasmid with the resistance/reporter marker gene cassette and plated on an organic medium containing 50 µg mL^−1^ hygromycin. Hygromycin-resistant colonies of *E. gracilis* were observed on selective plates.

The ATMT of *E. gracilis* with vector systems pWEN 100 and 200 yielded hygromycin-resistant colonies on a selective medium (Figure 3). No growth of *E. gracilis* cocultivated with *Agrobacterium* cells without the resistance/reporter gene cassette (control) was observed on a selective medium (Figure 3). These results indicated that the growth of *E. gracilis* on the selective medium is probably due to the expression of the marker *hptII* transferred into cells. The colonies growing in selection plates were randomly picked and transferred to a fresh organic medium containing hygromycin (50 µg mL^−1^). The transgenic lines were transferred at 20–25-day intervals to a new organic medium with identical composition and maintained on an agar medium. *Agrobacterium* contamination in the transgenic lines was checked by inoculating a loop-full of culture on a YEB agar medium and observing the growth of *Agrobacterium* at 28 °C. No colonies of *Agrobacterium* were observed on YEB plates up to seven days of incubation (data not shown), indicating that hygromycin-resistant *E. gracilis* cells are free from *Agrobacterium*, which was further affirmed by the PCR analysis as mentioned below.

### 2.3. Analysis of Transgene Integration and Expression

#### 2.3.1. PCR of Transformants

**Figure 4:**
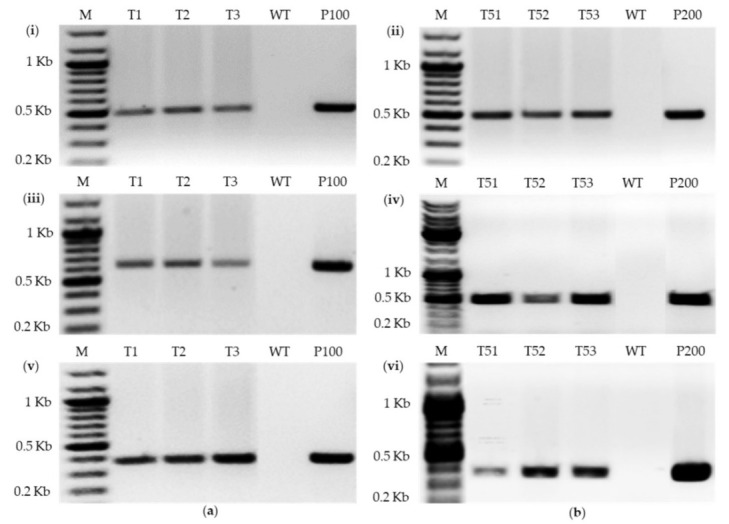
Confirmation of transgene insertion into *E. gracilis* cells. PCR-based confirmation for transforming (**a**) pWEN 100 and (**b**) pWEN 200 into *E. gracilis*. PCR analysis with *hptII* gene-specific primers (HptII-1839-For and HptII-1840-Rev) yielded a 495-bp product (**i**,**ii**), with *egfp* gene-specific primers (GFP-1847-For and GFP-1848-Rev)—a 609-bp product (**iii**), with *gus* gene-specific primers (GUS-1851-For and GUS-1852-Rev)—a 465-bp product (**iv**), and with CaMV35S promoter region primers (CaMV35S-1853-For and CaMV35S-1854-Rev)—a 382-bp product (**v**,**vi**). Lane labels stand for size markers (M), hygromycin-resistant cells hosting T-DNA of pWEN 100 (T1–T3) or pWEN 200 (T51–53), wildtype (WT) samples and the vectors pWEN 100 (P100) and pWEN 200 (P200) as positive controls.

The presence of the T-DNA region in the genome of putative transformants was checked by the PCR on the genomic DNA using different amplicons from the resistance marker gene *hptII*, the reporter genes *gus* or *gfp*, and the promoter CaMV35S. Genomic DNA was isolated from transgenic lines (see the Materials and Methods section), and a fragment of the resistance marker or the promoter or the reporter gene was amplified using primers as listed in Table 1. An expected amplicon with the size of 495 bp was obtained with the primers HptII-1839-For and HptII-1840-Rev specific to the *hptII* gene region in hygromycin-resistant clones (Figure 4a_i_,b_ii_). No products were amplified with the wildtype DNA (Figure 4a_i_,b_ii_). PCR with the *egfp* gene-specific primers (GFP-1847-For and GFP-1848-Rev) and *gus* gene-specific primers (GUS-1851-For and GUS-1852-Rev) yielded expected amplicons of 609 bp (Figure 4a_iii_) and 465 bp (Figure 4b_iv_), respectively, only in the transformants and positive control samples. Furthermore, reaction with the primers CaMV35S-1853-For and CaMV35S-1854-Rev specific to the CaMV35S promoter produced expected amplification products of 382 bp in all the hygromycin-resistant clones hosting T-DNA from pWEN 100 or pWEN 200 but not in the wild type (Figure 4a_v_,b_vi_). These results indicate the successful transfer and most likely integration of the complete T-DNA unit into *E. gracilis*.

To exclude the possibility of T-DNA region amplification due to *Agrobacterium* contamination, PCR was carried out for the *VirC* gene of the *Agrobacterium* Ti plasmid. A single band for the gene was only found in the positive control, while no amplification was observed in wildtype and hygromycin-resistant *E. gracilis* cells (Figure S4). This proved that the amplification of T-DNA parts is because of *E. gracilis* transformation and not due to *Agrobacterium* contamination.

#### 2.3.2. Expression of Reporter Genes

**Figure 5:**
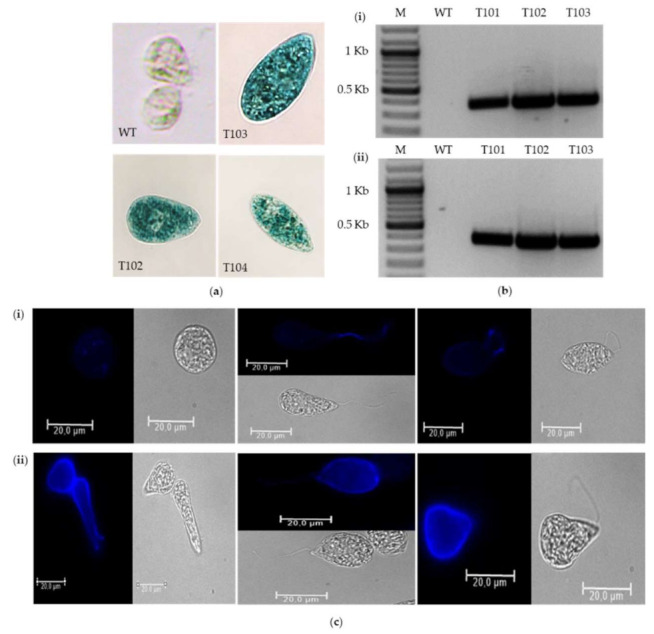
Analysis of transgene integration and expression in *E. gracilis*. (**a**) GUS histochemical assay of *E. gracilis* cells transformed with pWEN 650. Wildtype and hygromycin-resistant cells were treated with a substrate for β-glucuronidase, destained, and observed at a magnification of 400× for GUS expression. Transformed *E. gracilis* cells hosting the *hptII-2A-gus* gene stained blue (T101–T103) due to the activity of GUS, while blue color is absent in control *E. gracilis* cells (WT). (**b**) PCR-based confirmation for the transformation of pWEN 650 into *E. gracilis*. PCR analysis with *hptII* gene-specific primers (HptII-1841-For and HptII-1842-Rev) and *gus* gene-specific primers (GUS-1849-For and GUS-1850-Rev) yielded expected amplicons of 359 bp (**i**) and 377 bp (**ii**), respectively, only in transformants (T101–T103) and not in control wildtype *E. gracilis* cells (WT). The lane label M stands for size markers. (**c**) Indirect immunofluorescence assay of wildtype cells and transformants with the anti-GUS antibody. Wildtype *E. gracilis* cells (**i**) and *E. gracilis* cells transformed with the binary vector pWEN 650 (**ii**). Scale bar = 20 μm.

Transformation of *E. gracilis* with the pWEN 100 or pWEN 200 vector systems yielded hygromycin-resistant cells. However, hygromycin-resistant cells did not exhibit EGFP fluorescence or blue coloration as determined by the EGFP fluorescence and GUS histochemical assays, respectively (data not shown), although PCR amplification confirmed the presence of the *egfp* and *gus* genes. The hygromycin-resistant cells hosting the *hptII* and *egfp* genes did not emit the bright greenish-yellow fluorescence characteristic of the GFP. Furthermore, the transformants harboring the T-DNA region containing the *hptII* and *gus* genes of the vector pWEN 200 did not exhibit positive results in the GUS histochemical assay. This may indicate the possibility that the reporter genes, *egfp* and *gus*, had explicitly been silenced. Thus, we explored the potential of the FMDV 2A-coding sequence to link the reporter gene (i.e., *gus*) expression to that of the selection marker gene (i.e., *hptII*) in *E. gracilis* to circumvent transgene silencing. The *hptII-2A-gus* gene construct was placed under the control of the CaMV35S promoter and 5′UTR of the light-harvesting chlorophyll-binding protein of photosystem II (LHCPII) of *E. gracilis* in the vector pWEN 650 (Figure 2, Figure S3). The 5′UTR of LHCPII of *E. gracilis* was selected in this study because endogenous intronic sequences or UTRs in transgenes are known to enhance transgene stability and efficiency [29,37]. The construct was transformed into *E. gracilis* using the ATMT approach. The construct yielded hygromycin-resistant and GUS-positive cells (Figure 5a_i_). This suggests that the *hptII-2A-gus* ORF produced a functional hptII protein product that confers resistance to hygromycin and leads to a functional GUS protein product that can degrade the substrate X-Gluc and yield blue-stained cells.

The presence of the T-DNA region from the vector pWEN 650 in hygromycin-resistant, GUS-positive cells was confirmed by PCR. Amplicons of 359 bp and 377 bp with *hptII* gene-specific primers (HptII-1841-For and HptII-1842-Rev) and *gus* gene-specific primers (GUS-1849-For and GUS-1850-Rev), respectively, were successfully detected from the *E. gracilis* cells transformed with pWEN 650 (Figure 5b). The presence of gene-specific fragments in transgenic lines with no amplicons detected in the wildtype *E. gracilis* indicates the successful transfer of both the resistance and reporter marker genes as a single T-DNA unit flanked by the T-DNA left and right borders. An indirect immunofluorescence assay was also performed to monitor the distribution of the transgene product (GUS). The indirect immunofluorescence assay showed that GUS is scattered all over the transformed cell, suggesting cytosolic localization (Figure 5c_ii_). A strong GUS signal was observed along the membrane of transformants, whereas no signal was detected for the wildtype *E. gracilis* (Figure 5c_i_).

### 2.4. Optimization of Factors in the ATMT Procedure of E. gracilis

**Figure 6:**
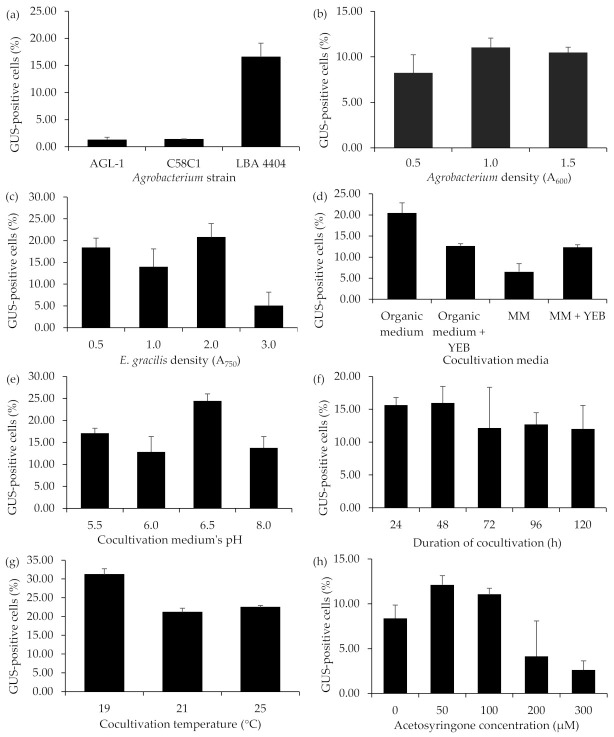
The effects of different cocultivation parameters on ATMT of *E. gracilis*. (**a**) *A. tumefaciens* strain; (**b**) *Agrobacterium* concentration; (**c**) *E. gracilis* concentration; (**d**) cocultivation media; (**e**) pH of the cocultivation medium; (**f**) cocultivation duration; (**g**) cocultivation temperature; (**h**) acetosyringone concentration. The values are the means ± standard deviation of three independent experiments.

The effect of different cocultivation parameters (*Agrobacterium* strains, bacterial and *E. gracilis* concentrations, cocultivation duration, temperature, cocultivation medium, pH of the medium, and acetosyringone concentration) on the transformation efficiency of *E. gracilis* was evaluated with the pWEN 650 vector. The transformation rate was determined by calculating the percentage of GUS-expressing cells as analyzed by the GUS histochemical assay [48]. Initially, the efficacy of three *Agrobacterium* strains, LBA4404, C58C1, and AGL-1, to transfer T-DNA into the genome of *E. gracilis* was tested. Based on other microalgae species transformation [35,36,44,45], the *Agrobacterium* culture’s optical density was kept at 1.0 (A_600_). The optical density of *E. gracilis* was set to 2.0 (A_750_). The bacterial strains were cocultivated with algal cells in an organic medium (pH 6.5) at 25 °C for two days. The *Agrobacterium* strain LBA4404 produced 15 times more GUS-stained cells than C58C1 and AGL-1 (Figure 6a). Thus, all the following experiments on optimizing cocultivation parameters were performed with the *A. tumefaciens* strain LBA4404.

Among the tested cell concentrations of *Agrobacterium* (A_600_: 0.5, 1.0, and 1.5) and *E. gracilis* (A_750_: 1.0, 1.5, 2.0, and 3.0), *Agrobacterium* and *Euglena* cell densities of 1.0 (A_600_) and 2.0 (A_750_), respectively, were found to be ideal for generating higher numbers of GUS-positive cells (Figure 6b,c). Four different media were tested to determine the optimal cocultivation medium to obtain higher numbers of transformants: a mineral medium (MM) [49], an organic medium, and a 1:1 mixture of an organic medium or MM with the YEB *Agrobacterium* growth medium. Although all of these media favored good growth of both the *Agrobacterium* (Figure S5a) and *E. gracilis* cells (Figure S5b), the cocultivation in the organic medium yielded a higher number of transformants. In this medium, the transformation efficiency was eight times higher than in the MM medium (Figure 6d). Among the four different pH values (pH 5.5, 6.0, 6.5, and 8.0) of the organic medium evaluated, higher numbers of GUS-positive cells were noted at pH 6.5 (Figure 6e). No significant effect in the growth behavior of *E. gracilis* and the *A. tumefaciens* strain LBA4404 was observed in the organic medium with pH values 5.5, 6.0, 6.5, and 8.0 (Figure S6a,b).

Among the tested cocultivation periods (24, 48, 72, 96, and 120 h), a 48-h incubation period was optimal for obtaining a higher yield of GUS-positive cells (Figure 6f). Lower transformation efficiency was noted with a longer cocultivation period. Initial studies showed that temperatures > 25 °C had a dramatic effect on the growth of wildtype *E. gracilis* (Figure S7). The study on the impact of three different cocultivation temperatures (viz. 19, 21, and 25 °C) on the transformation rate showed that higher numbers of GUS-positive cells were achievable at 19 °C (Figure 6g). Cocultivation at 21 and 25 °C resulted in a sequential drop in blue-colored cells, suggesting that lower temperatures favor *E. gracilis* transformation. Acetosyringone concentrations up to 500 µM did not show any inhibitory effect on the growth of *E. gracilis* in the organic medium (Figure S8). Cocultivation performed with 50 µM acetosyringone yielded the highest transformation rate compared to the other tested concentrations (0, 100, 200, and 300 µM). A decrease in the number of transformants was noted with an increase in the acetosyringone concentration (Figure 6h).

Collectively, the transformation efficiency increased using the *Agrobacterium* strain LBA4404 in the organic medium with a pH value of 6.5. The *Agrobacterium* density should be around 1.0, whereas the density of the algal cells should be 2.0. The transformation should occur at 19 °C in the presence of 50 µM acetosyringone for no longer than two days to yield a high transformation efficiency.

### 2.5. Genetic Stability of Transformed Clones

The persistence of the hygromycin-resistant phenotype was checked to demonstrate the stability of transgenic *Euglena* cells. The transformed cells were transferred from the organic medium with hygromycin (50 µg mL^−1^) followed by cultivation in a hygromycin-free medium, and their growth was monitored. This culturing process was performed every 25–30 days for more than a year. It is remarkable that even in the absence of antibiotic selection, hygromycin resistance marker gene silencing was not observed. All the transformed cells grew in the medium containing hygromycin (50 µg mL^−1^) even after transfer from the hygromycin-free medium, indicating that the transformed cells’ growth for subsequent generations is due to the stable expression of the *hptII* gene. This characteristic is also apparent from the amplification of T-DNA regions from genomic DNA of transformants taken at different time intervals (data not shown). The oldest clones were transferred more than 30 times with the retention of the hygromycin-resistant phenotype.

## 3. Discussion

Genetic engineering has emerged as a powerful strategy for improving natural product biosynthesis and producing specific compounds in engineered heterologous hosts in quantities sufficient for industrial applications. However, molecular tools for the genetic engineering of a majority of microalgal strains are lacking. Establishing toolkits for the genetic engineering of biotechnologically important microalgal species is essential to opening up new opportunities for microalgae industrialization.

In the genetic engineering of microalgae, the selection and transformation procedures are necessary steps to accomplish efficient genetic modification. Selectable marker genes that encode proteins that confer resistance to antibiotics are an essential part of cloning vectors; putative transformants need to be distinguished from non-transformants. Several authors have reported the use of antibiotics, such as hygromycin, kanamycin, zeocin, chloramphenicol, erythromycin, spectinomycin, paromomycin, neomycin, and G418, for the selection of recombinant microalgae strains [33,37,42,50,51]. The antibiotic sensitivity analysis showed that concentrations ≥ 30 µg mL^−1^ of the antibiotics hygromycin and zeocin completely inhibited *E. gracilis* growth in both liquid and solid media. In contrast, the tested concentrations of the antibiotic kanamycin did not show any growth inhibitory effects on the culture. Nakazawa et al. [33] have also reported using zeocin at 20–100 µg mL^−1^ and hygromycin at 5–100 µg mL^−1^ for the section of antibiotic-resistant *E. gracilis*. The antibiotics ticarcillin/clavulanate and cefotaxime have been used to eliminate *Agrobacterium* cells from the microalgae cells after transformation [35,40,41,44,45,52]. Thus, the toxicity of these antibiotics on *E. gracilis* cells has been tested. The results demonstrate that both antibiotics do not affect the growth of *E. gracilis*. This indicates the possibility of applying ticarcillin/clavulanate or cefotaxime to remove *Agrobacterium* cells from the *Euglena* culture after cocultivation.

Therefore, our data are essential not only for selecting transformants but also for maintaining axenic cultures. Due to the high sensitivity of *E. gracilis*, the antibiotics hygromycin and zeocin would be suitable as selection markers in transformation. The antibiotics kanamycin, ticarcillin/clavulanate, and cefotaxime that did not exhibit any growth inhibition could be used at the standard concentration to eliminate bacterial contaminants from the *E. gracilis* culture. In this study, ticarcillin/clavulanate at the concentration of 500 µg mL^−1^ was used to eliminate *Agrobacterium* cells after the cocultivation of *E. gracilis* and *Agrobacterium*. Hygromycin at the concentration of 50 µg mL^−1^ was selected as a suitable antibiotic for the genetic transformation of *E. gracilis* since hygromycin exhibited strong growth inhibitory effects and is inexpensive. Besides, the *hptII* gene conferring resistance to the antibiotic hygromycin has been used to transform several microalgal species [35,39,40,42,51].

The transformation of *E. gracilis* with the ATMT approach [36,44,45] yielded hygromycin-resistant *E. gracilis* colonies, indicating the transfer and expression of the T-DNA region. The PCR analysis further confirmed the presence of the T-DNA region gene in transformants. Hygromycin-resistant *E. gracilis* colonies with the ATMT method support the fact that T-DNA is integrated with the help of the associated proteins expressed before the infection by *A. tumefaciens* [47,53]. The possibility of *Agrobacterium* presence in transformants was ruled out by the PCR analysis of the *VirC* gene of the *Agrobacterium* Ti plasmid and by detecting the growth of *Agrobacterium* on the YEB agar medium inoculated with a transgenic culture.

Although the PCR analysis confirmed the *egfp* and *gus* genes’ presence, the hygromycin-resistant cells hosting the *hptII* and either of the reporter genes (*gus* or *egfp*) did not exhibit positive results in the GUS histochemical assay and the EGFP analysis. This may indicate that the reporter genes, *egfp* and *gus,* had explicitly been silenced. Silencing of transgenes has been generally suggested in microalgae [35,54,55,56]. Neupert et al. [57] attributed the robust gene silencing mechanism(s) to the low level of GFP expression from the nuclear genome in *Chlamydomonas*. Cha et al. [35] also reported silencing the *egfp–gusA* fusion gene in *Chlorella vulgaris* (*C. vulgaris*) transformed via the *A. tumefaciens* LBA4404 pCAMBIA vector. Zaslavskaia et al. [58] suggested partial inactivation of the integrated plasmid as the cause for the lack of reporter gene expression in half of the antibiotic-resistant diatom *Phaeodactylum tricornutum*. Silencing of the *gus* gene and not of the bleomycin resistance marker gene (*ble*) has also been observed in some *Tetraselmis chuii* (*T. chuii*) transformants in successive generations [40]. Úbeda-Mínguez et al. [40] pointed out that since some of the silencing mechanisms do not necessarily rely on chromatin packaging effects, it is possible that the *gus* gene was specifically silenced while the *ble* gene is active, conferring phleomycin resistance to *T. chuii* cells. It might also be that the reporter gene mRNA in *E. gracilis* might be specifically targeted for degradation as it has been indicated that posttranscriptional gene silencing involves sequence-specific degradation of transgene RNA and is typically observed when foreign transgenes are introduced under the control of a strong CaMV35S promoter [59]. It can also be speculated that the lack of reporter proteins (GUS or EGFP) in hygromycin-resistant *E. gracilis* cells is due to the mutual interference of the CaMV35S promoters located in close vicinity, controlling the expression of the resistance and reporter marker genes.

Furthermore, a recent analysis of the *E. gracilis* transcriptome has shown the presence of components of the RNA-mediated gene-silencing machinery: Dicer-like, which cleaves double-stranded RNA [60], and Argonaute, which targets the inactivation of sequences complementary to small RNAs (21–24 nucleotides) as part of the RNA-induced silencing complex [11,61]. Other components of the gene-silencing machinery, such as helicases, histone methylases, histone deacetylases, and cytosine methylases, are also noted in the *Euglena* transcriptome [11]. Therefore, there might be several mechanisms behind gene silencing in *E. gracilis* transformants. Thus, strategies to improve transgene expression and circumvent silencing in the nuclear genome were investigated. It has been shown that coupling the gene of interest with a resistance marker gene using the FMDV 2A peptide helps to circumvent transgene silencing and increases the production of the protein of interest in microalgae [62,63,64]. The FMDV 2A peptide is a 20-amino acid sequence that mediates the self-cleavage reaction [65,66]. Rasala et al. [63] fused the resistance gene *ble* to the GFP gene via the FMDV 2A linker peptide and demonstrated its application to obtain zeocin-resistant *Chlamydomonas reinhardtii* (*C. reinhardtii*) strains accumulating high levels of GFP. The same system was also used to successfully express six fluorescent proteins in *C. reinhardtii* [64]. The inclusion of endogenous intronic sequences or untranslated regions (UTRs) in transgenes has been shown to enhance heterologous gene expression’s stability and efficiency [62,67,68,69]. In this study as well, the reporter gene *gus* fusion with the resistance marker gene *hpt II* using the FMDV 2A peptide and inclusion of the 5′UTR of the LHCPII of *E. gracilis* in the insert yielded transformants exhibiting hygromycin resistance as well as producing a functional GUS protein product. A difference in blue fluorescence in the indirect immunofluorescence assay was observed between the transformants. This might be due to the differences in the T-DNA insertions copy number and/or position effects [44,45,46,47]. The integration of the T-DNA gene cassette in the transcriptionally active region in the transformant exhibiting strong blue fluorescence could have led to a higher and stable expression of the *gus* gene, resulting in a high degree of blue fluorescence than in transformants exhibiting weak fluorescence. 

The parameters known to influence the efficiency of the ATMT include *Agrobacterium* strains, plasmids, bacterial concentration, preculture duration, chemical inducers, and cocultivation duration, temperature, medium, and the medium’s pH [35,36,38,40,44,45]. The *Agrobacterium* strain’s infection ability is considered an important factor influencing transformation frequency. Strain LBA4404 has been used to transform several microalgal species successfully [35,36,44,45]. Cheng et al. [43] observed that the aggregates of *Schizochytrium* infected by *A. tumefaciens* LBA4404 were much larger than those infected by EHA105, suggesting that LBA4404 has a higher binding affinity to *Schizochytrium* cells. Recently, Srinivasan and Gothandam [70] also reported the highest transformation rate with LBA4404 (181 ± 3.78 CFU 10^–6^ cells), followed by GV3101 (128 ± 5.29 CFU 10^–6^ cells) and EHA105 (61 ± 5.03 CFU 10^–6^ cells) in *Dunaliella* transformation. In this study as well, higher numbers of *E. gracilis* transformants were obtained with the *A. tumefaciens* strain LBA4404 compared to C58C1 and AGL-1. The concentration of *Agrobacterium* and *Euglena* cells was fixed to 1.0 (A_600_) and 2.0 (A_750_), respectively, which were found to be the optimal cocultivation densities to obtain higher numbers of transformants. Cha et al. [35] also observed an increase in the percentage of GUS-positive *Chlorella* cells with the increase in bacterial density from 6.6% (A_600_ = 0.2) to 15.1% (A_600_ = 1.0), suggesting A_600_ = 1.0 as optimum for the *A. tumefaciens* culture. For *E. gracilis* transformation, an organic medium with a pH 6.5 was considered the ideal cocultivation medium. This aligns with the findings reporting an acidic pH to transform microalgal species since the acidic environment induces *Agrobacterium* virulence gene expression [35,40,43].

The cocultivation period affects transformation efficiency both in plants [71,72] and microalgae species [35,36,44,45]. In the case of *E. gracilis* transformation, an incubation period of 48 h was found to be optimal, which is also observed to be suitable for the ATMT of *C. reinhardtii* [36,38,73], *Haematococcus pluvialis* (*H. pluvialis*) [39], and *Isochrysis galbana* [36]. A longer cocultivation period might have led to an overgrowth of the *Agrobacterium* and the host cells’ necrosis and death [72,74]. It is evident from previous studies that cocultivation temperature influences the transformation efficiency and that the optimum temperature for T-DNA delivery and stable transformation differs with each *Agrobacterium* strain and each specific explant or host cell [35,40,46]. A higher number of GUS-positive *E. gracilis* cells was observed at 19 °C, contrasting with other microalgae species’ findings. A temperature of 27 °C is optimum for *T. chuii* transformation [40], which is relatively higher than that reported for *C. vulgaris* (24–25 °C) [35], *Isochrysis* sp. (25 °C) [36], *Chlamydomonas* (23 °C) [73], and *H. pluvialis* (22 °C) [39]. It has been observed that *Agrobacterium* infection of *Chlamydomonas* [73] and *Haematococcus* [39] is feasible without the inclusion of acetosyringone. However, the inclusion of phenolic compounds (e.g., acetosyringone) dramatically enhances transformation efficiency [35,36,44,45]. In this study as well, the transformation efficiency was affected by the inclusion of acetosyringone. Higher levels of transformants were obtained in the presence of acetosyringone than in the absence. However, concentrations above the optimum (50 µM) yielded lower numbers of transformants.

The ATMT of *E. gracilis* conducted under the optimized cocultivation parameters yielded a transformation efficiency of 8.26 ± 4.9%, higher than that reported very recently for *E. gracilis* (0.1%) [29]. Lower transformation frequencies have also been reported for other microalgae species, such as *Dunaliella* (0.004%) [52], *H. pluvialis* (0.0153%) [39], *C. reinhardtii* (0.052%) [38], and *Isochrysis* sp. (0.85%) [36]. Nevertheless, a transformation efficiency of 25% has also been reported in *Chlorella* cells transformed by *A. tumefaciens* strain LBA4404 harboring pCAMBIA 1304 under the optimized cocultivation parameters [35].

## 4. Materials and Methods

### 4.1. Growth Conditions of E. gracilis

The microalga *E. gracilis* KLEBS strain Z was obtained from the algal culture collection center at the University of Göttingen, Germany [75]. It was grown in an organic medium [76] at 21 °C and under continuous low-light conditions (22 W m^−2^) in static Erlenmeyer flask cultures.

### 4.2. Vector Systems and Bacterial Strains

A schematic map of different gene cassettes cloned in binary vector systems is shown in Figure 2 and Figure S3. The binary vector pWEN 100 hosting a hygromycin resistance marker gene (*hptII*) and a reporter gene (e*gfp*), both driven by the CaMV 35S promoter, was kindly provided by Prof. Benedikt Kost, University of Erlangen–Nuremberg, Germany. It was used as the backbone vector for cloning different gene cassettes to obtain the binary vectors pWEN 200 and pWEN 650 (Figure 2, Figure S3). In pWEN 200, the reporter gene e*gfp* was replaced with the reporter gene *gus*. The *gus* gene with the *Kpn*I and *Sac*I enzyme sites at the ends was amplified from pBK17 (Benedikt Kost, University of Erlangen–Nuremberg) using the GUS-forward and -reverse primers (Table 1) and cloned into pWEN 100 at the respective enzyme sites to obtain pWEN 200. The binary vector pWEN 650 was constructed by joining DNA segments through overlap extension PCR as follows. Segment 1 containing the 5′UTR of the LHCPII (GenBank accession number X61361.1) was amplified from the *E. gracilis* genomic DNA with the primers LHP5UTR-forward and -reverse (Table 1). The forward primer provided a *Sma*I restriction site. Segment 2 with the hygromycin resistance gene interrupted by the first intron of LHCPII was amplified using the LHPINT-forward and -reverse primers (Table 1). Segment 3, the *gus* reporter gene with a FMDV 2A (F2A) linker sequence at the 5′ end, was amplified with the F2AGUS-forward and -reverse primers (Table 1). Segment 4 containing the 3′UTR of the LHCPII was amplified from the *Euglena* genomic DNA with the LHP3UTR primers containing a *Sac*I restriction site within the reverse primer (Table 1). After assembling PCR segments 1 and 2 through overlap extension PCR, the product was cloned into a pGEM-Teasy vector (Promega GmbH, Mannheim, Germany). The assembled segment was then joined with segment 3 and segment 4 using a similar approach and subcloned into a pGEM-T easy vector. The complete fragment 1–4 was digested with *Sma*I and *Sac*I and then cloned into pWEN 100 linearized with the same enzymes. All the restriction enzymes were purchased from NEB (Ipswich, Cambridge, MA, USA).

*Escherichia coli* strain DH5α used to maintain and propagate vector systems was grown in the Luria–Bertani (LB) medium at 37 °C. The three *A. tumefaciens* strains LBA4404, AGL-1, and C58C1 used for *E. gracilis* transformation were cultured in the YEB *Agrobacterium* growth medium supplemented with suitable antibiotics at 28 °C. The binary vectors were transferred into the *Agrobacterium* strains using a freeze-thaw protocol [77].

### 4.3. Sensitivity of Microbial Strains to Antibiotics

The sensitivity of *E. gracilis* to the antibiotics hygromycin (0, 10, 30, 40, 50, 80, or 100 µg mL^−1^) (Carl Roth, Karlsruhe Germany), zeocin (0, 15, 30, 50, 75, or 100 µg mL^−1^) (ThermoFischer Scientific, Cambridge, MA, USA), kanamycin (0, 20, 50, 100, or 200 µg mL^−1^) (Carl Roth, Karlsruhe Germany), cefotaxime (0, 250, or 500 µg mL^−1^) (Merck KGaA, Darmstadt, Germany), and ticarcillin 2NA and clavulanate K (0, 250, or 500 µg mL^−1^) (Duchefa Biochemie, Haarlem, the Netherlands) was tested in an organic medium. *E. gracilis* was inoculated at a final concentration of 0.2 (absorbance at 750 nm (A_750_)) in an organic medium containing the antibiotic and incubated in a culture chamber under continuous light at 22 W m^−2^ and 21 °C. The growth rate of the culture was monitored by multiple measurements of absorbance at 750 nm and regular intervals using a spectrophotometer. To determine the sensitivity of *E. gracilis* to antibiotics in an agar medium, 1.0 × 10^5^ cells mL^−1^ were spread inoculated on organic agar plates supplemented with varying concentrations of the antibiotics as mentioned above and incubated under continuous light at the irradiance of 22 W m^−2^ and the temperature of 21 °C. The growth of *E. gracilis* on a solid medium was monitored daily for 3–4 weeks. The sensitivity of *Agrobacterium* strains LBA4404, AGL-1, and C58C1 to varying concentrations of ticarcillin/clavulanate (0, 250, 500, or 1000 µg mL^−1^) or cefotaxime (0, 250, 500, or 1000 µg mL^−1^) was tested in the YEB liquid medium. The antibiotics were added to the freshly grown cells of *Agrobacterium* and incubated at 28 °C and 200 RPM. After 24 h of incubation, *Agrobacterium* strains’ growth was assayed by measuring the culture absorbance at 600 nm. Each antibiotic test was repeated at least three times.

### 4.4. Transformation of Euglena

Transfer of gene cassettes into *E. gracilis* was done using the ATMT approach [36,44,45]. Late log-phase cells of the *E. gracilis* and *A. tumefaciens* strain LBA4404 hosting vector system were harvested, suspended in the organic medium in a tube, and incubated at 21 °C. The final concentration of *A. tumefaciens* and *E. gracilis* cells in the cocultivation medium was kept at 1.0 (A_600_) and 2.0 (A_750_), respectively. After cocultivation, the cells were centrifuged at 3000× *g* for 5 min, spread-inoculated on organic medium plates containing lethal concentrations of hygromycin (50 µg mL^−1^) and ticarcillin/clavulanate (500 µg mL^−1^) as determined by the antibiotic sensitivity assay, and incubated at 21 °C under continuous light at 22 W m^−2^. The negative control included the set of *E. gracilis* cells cocultivated with the *A. tumefaciens* strain transformed with a plasmid lacking resistant reporter gene cassettes. The hygromycin-resistant colonies that appeared after 3–4 weeks were randomly picked and transferred into the organic medium containing ticarcillin/clavulanate (500 µg mL^−1^), which was added to eliminate *Agrobacterium*.

### 4.5. Confirmation of A. tumefaciens’ Absence in Hygromycin-Resistant E. gracilis

Genomic DNA from wildtype and hygromycin-resistant *E. gracilis* was isolated using PureLink™ Genomic DNA Mini Kit (K182001) (ThermoFischer Scientific, Cambridge, MA, USA). To confirm the absence of *A. tumefaciens* in the purified (ticarcillin/clavulanate-treated) hygromycin-resistant *E. gracilis* cells, PCR was performed with the primers specific to the *VirC* gene of *A. tumefaciens* (VirC-forward and -reverse primers) (Table 1). The *A. tumefaciens* Ti plasmid was used as the positive control for the *VirC* gene, while genomic DNA from wildtype *E. gracilis* was used as the negative control. The PCR products were separated on 1% agarose gel and documented using an electrophoresis gel documentation system. Detection of contaminating *Agrobacterium* in ticarcillin/clavulanate-treated hygromycin-resistant *E. gracilis* cells was also performed by inoculating an aliquot of culture on a YEB agar plate and monitoring the growth of *Agrobacterium* at 28 °C for a week.

### 4.6. PCR Analysis of Transformants

For the PCR analysis, wildtype cells and transformants’ genomic DNA was isolated using PureLink™ Genomic DNA Mini Kit (K182001) (ThermoFischer Scientific, Cambridge, MA, USA). The primers used to amplify different parts of the T-DNA region are listed in Table 1. Amplification was carried out in a thermal cycler (C1000 Touch, Bio-Rad, Hercules, CA, USA). The PCR profile included 95 °C for 30 s followed by 29 cycles of amplification at 95 °C for 20 s, 60 °C or 68 °C for 20 s, and the final extension at 68 °C for 5 min. The amplified products were separated on 1% agarose gel and documented using an electrophoresis gel documentation system.

### 4.7. Analysis of Reporter Genes Expression in Hygromycin-Resistant Cells

#### 4.7.1. EGFP Fluorescence Analysis

Hygromycin-resistant and control (wildtype) *Euglena* cells were collected after centrifugation at 3000× *g* for 5 min. EGFP fluorescence was observed using an inverted microscope (DMI4000B; Leica, Wetzlar, Germany) with an X-Cite 200DC fluorescence illuminator and a cooled digital black-and-white camera (DFC 365 FX) with HCX PL FLUOTAR L 403/0.60 lenses. The excitation wavelength was kept at 488 nm, and the emission was recorded with a long pass filter of 500 nm.

#### 4.7.2. Determination of GUS Activity

The GUS histochemical assay of wildtype and hygromycin-resistant *Euglena* cells was performed as follows. The cells were collected by centrifugation at 3000× *g* for 5 min and washed with 0.5 mL of 200 mM phosphate buffer (pH 7) containing 4 mM EDTA. After washing, the cells were suspended in 0.5 mL GUS staining solution (50 mM NaH_2_PO_4_, 50 mM Na_2_HPO_4_, 5 mM K_3_Fe(CN)_6_, 5 mM K_4_Fe(CN)_6_, 3 mg X-Gluc (5-bromo-chloro-indolyl glucuronide cyclohexylammonium salt, Sigma-Aldrich Co., St. Louis, LA, USA), 5 mL Triton X-100, pH 7.0) [48]. The suspended cells were incubated overnight at 37 °C in the dark. After incubation, the cells were collected by centrifugation at 3000× *g* for 5 min to remove the staining solution and subsequently washed twice with 0.5 mL of 70% ethanol. Following washing, the cells were observed under a light microscope (Biozero BZ-8000K, Keyence, Osaka, Japan) at a 400× magnification. Blue staining was clear in transformants, but it was light green in the non-transformed controls.

### 4.8. Indirect Immunofluorescence Assay

The indirect immunofluorescence assay was performed using the standard protocol [78]. The primary antibody was the rabbit anti-GUS antibody (1:100, Agrisera), whereas the secondary antibody was the anti-rabbit IgG fluorophore-conjugated Alexa 488 antibody. Fluorescence was observed with a Leica DMI4000 B microscope and a Leica DFC365 FX camera and edited using the LAS AF Lite Software (Keyence, Osaka, Japan). The secondary antibody was excited at 488 nm and imaged between 500 and 550 nm. Image acquisition parameters were kept identical for the comparison of wildtype cells (control) and transformants.

### 4.9. Optimization of Transformation Parameters

The evaluated cocultivation parameters that are known to influence transformation frequency included *Agrobacterium* strains (LBA4404, AGL-1, and C58C1), *Agrobacterium* cell concentration (A_600_: 0.5, 1.0, and 1.5), *E. gracilis* cell concentration (A_750_: 1.0, 1.5, 2.0, and 3.0), cocultivation media (organic medium, organic medium + YEB (1:1), MM, and MM + YEB (1:1)), cocultivation time (one, two, three, four, and five days), cocultivation temperature (19, 21, and 25 °C), pH of the cocultivation medium (5.5, 6.0, 6.5, and 8.0) and acetosyringone concentration (0, 50, 100, and 200 µM). Based on the preliminary experiments’ findings, one parameter was varied while others were kept constant to evaluate each cocultivation condition’s effect on transformation. All the parameters were optimized via screening for the GUS expression. Following cocultivation, the cells were treated with 500 mg L^−1^ ticarcillin/clavulanate for 48 h to eliminate *Agrobacterium*. After ticarcillin/clavulanate treatment, the expression of the *gus* reporter gene was confirmed by the GUS histochemical assay [48]. Transformation frequency was determined by calculating the percentage of GUS-expressing cells in a Thoma chamber (VWR, Radnor, PA, USA) at 400× magnification under a light microscope (Biozero BZ-8000K, Keyence, Osaka, Japan). Each cocultivation parameter was examined in triplicate in at least three independent experiments.

### 4.10. Maintenance and Stability

The *Agrobacterium*-free *E. gracilis* transformants were transferred to the organic medium with an initial cell density of 0.2 (A_750_). The cultures were routinely transferred to a fresh organic medium at 20–25-day intervals. The transgenic cells were also maintained in the organic medium containing 50 µg mL^−1^ hygromycin with regular subculturing for over two years. *E. gracilis* transgenic lines’ stability was determined by transferring cultures from the antibiotic-free medium to a medium containing 50 µg mL^−1^ hygromycin and observing their growth. These culturing steps were repeated at different intervals for one year to analyze the genetic stability of transformants.

## 5. Conclusions

In conclusion, we report biotechnological tools that can be utilized for genetic modifications in the freshwater flagellate *E. gracilis*. Cocultivation of cells of *E. gracilis* and *A. tumefaciens* hosting binary vectors yielded transgenic *E. gracilis* cells expressing the marker and reporter genes. The transformation was confirmed by the PCR and GUS activity, as well as by indirect immunofluorescence assays. The established ATMT system opens the way to exploit *E. gracilis* for fundamental studies and biotechnological applications.

## Figures and Tables

**Table 1:** List of primers used in this study.

Sr. No.	Primer Name	Primer Sequence	Fragment Size (bp)
1	Gus-For	5′G***GGTACC***CGGGTGGTCAGTC3′	1893
	Gus-Rev	5′CGAGCTCGGTAGCAATTCCCG3′	
2	LHP5UTR-For	5′GACCTGCAAGAT***CCCGGG***cccagggaaacgtctctt3′	493
	LHP5UTR-Rev	5′ctttcatccactcacttcaaaATGAAAAAGCCTgagc3′	
3	LHPINT-For	5′ctttcatccactcacttcaaaATGAAAAAGCCTgagcacaactgccttcagacttg3′	1145
	LHPINT-Rev	5′CTCGTCCGGGATCTTGGCGGGTGAAACAGACTTTG3′	
4	F2AGUS-For	5′CTCGTCCGGGATCTTGGCGGGTGAAACAGACTTTGAATTTTGACCTTCTCAAGTTGGCGGGAGACGTGGAGTCCAACCCTGGACCTATGTTACGTCCTGTAGAAAC3′	1895
	F2AGUS-Rev	5′GCAGCAGGGAGGCAAACAATGA3′	
5	LHP3UTR-For	5′GGAGGCAAACAATGAtgatgtggacaacgcaact3′	410
	LHP3UTR-Rev	5′agcaactacggctagaagtAGGCCTACTAGT***GAGCTC***G3′	
6	HptII-1839-For	5′GACAGCGTCTCCGACCTG3′	495
	HptII-1840-Rev	5′CCAAAGCATCAGCTCATCG3′	
7	HptII-1841-For	5′GCGGTCATTGACTGGAGC3′	359
	HptII-1842-Rev	5′CGTCGGTTTCCACTATCGG3′	
8	GFP-1847-For	5′GACGTAAACGGCCACAAGTT3′	609
	GFP-1848-Rev	5′GAACTCCAGCAGGACCATGT3′	
9	GUS-1851-For	5′TGAAGATGCGGACTTACGTG3′	465
	GUS-1852-Rev	5′TGAGCGTCGCAGAACATTAC3′	
10	GUS-1849-For	5′CGACGCTCACACCGATAC3′	377
	GUS-1850-Rev	5′GGTCGCGAGTGAAGATCC3′	
11	CaMV35S-1853-For	5′CGCAGCAGGTCTCATCAAG3′	382
	CaMV35S-1854-Rev	5′GACAAGTGTGTCGTGCTCC3′	
12	VirC-For	5′ATCATTTGTAGCGACT3′	730
	VirC-Rev	5′AGCTCAAACCTGCTTC3′	

Restriction sites are in bold italics; For and Rev stand for forward and reverse.

## Data Availability

Not applicable.

## Supplementary Materials

The following are available online at https://www.mdpi.com/article/10.3390/ijms22126299/s1.
