# Supplementary material for: Agrobacterium tumefaciens-Mediated Nuclear Transformation of a Biotechnologically Important Microalga—Euglena gracilis
Source: Int J Mol Sci. 2021 Jun 11;22(12):6299. doi: 10.3390/ijms22126299 (PMC8230907; doi:10.3390/ijms22126299)
Supplement: Supplementary file 1 [file ijms-22-06299-s001.zip › ijms-1219398-supplementary.pdf]

Supplementary data

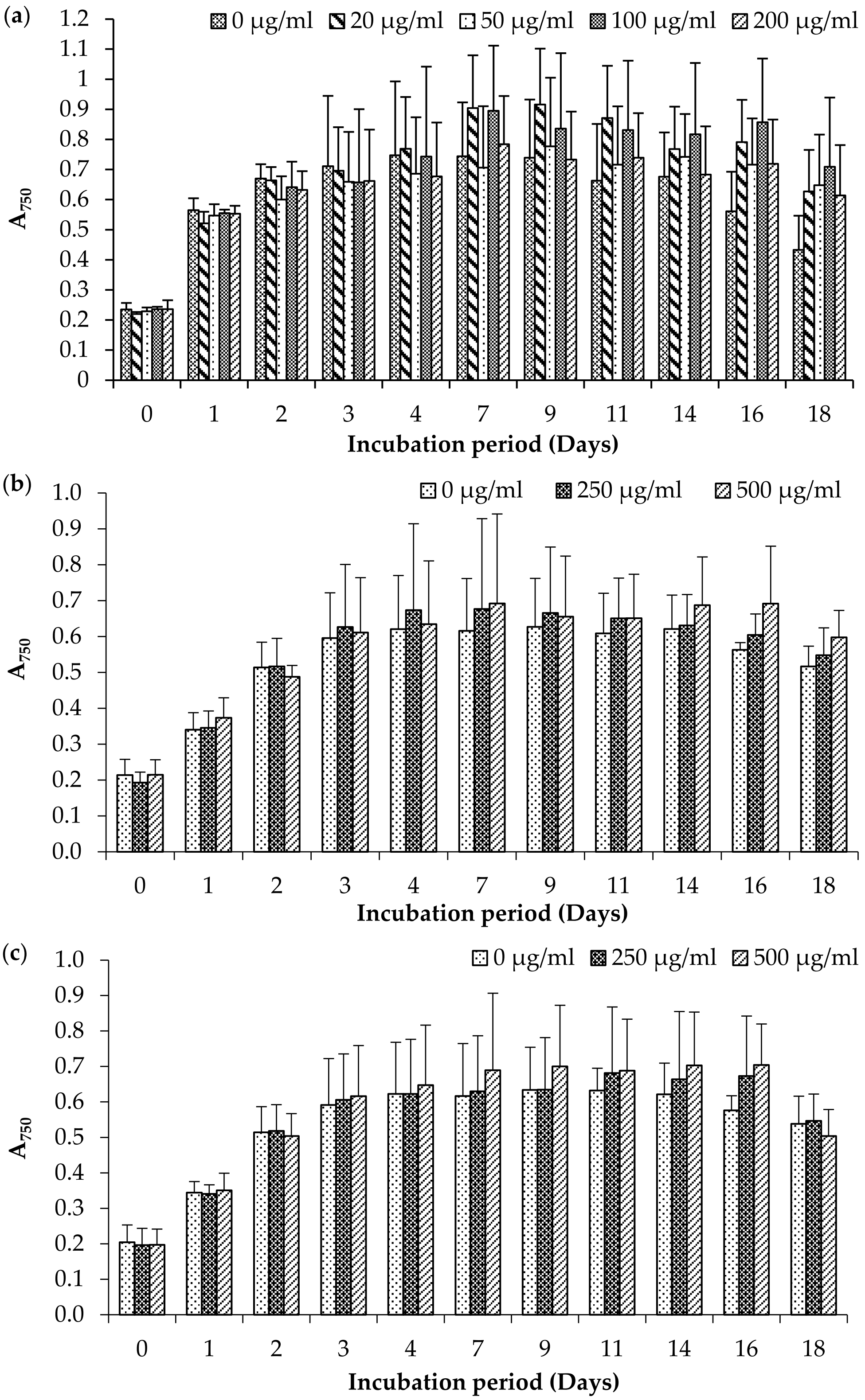

Figure S1. Growth inhibitory effects of different antibiotics on *E. gracilis*. (**a**) kanamycin (0, 20, 50, 100, and 200 μg mL^{-1}), (**b**) ticarcillin (0, 250, and 500 μg mL^{-1}), and (**c**) cefotaxime (0, 250, and 500 μg mL^{-1}). The growth of *Euglena* was assayed by measuring the culture absorbance at 750 nm. Each value indicates the mean ± standard deviations of three replicates.

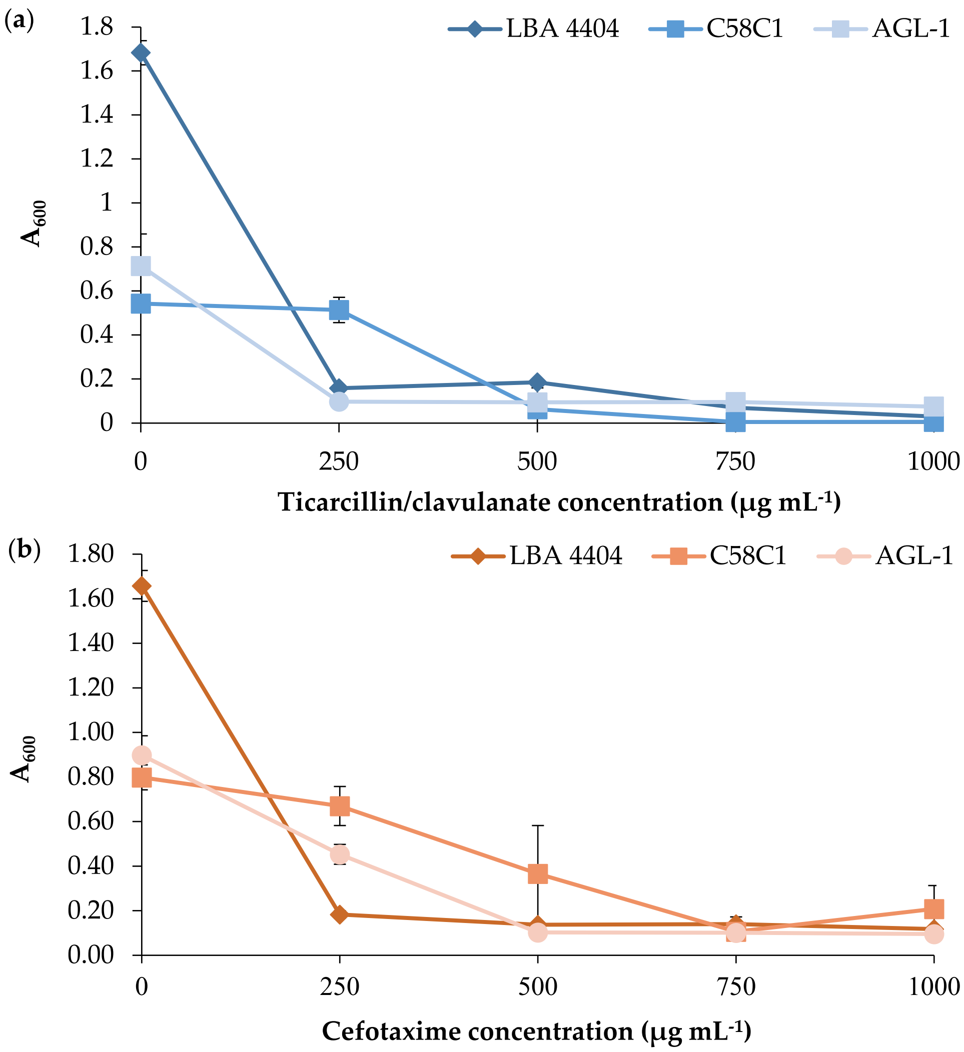

Figure S2. Growth sensitivity of *A. tumefaciens* strains LBA 4404, C58C1, and AGL-1 to the antibiotics (**a**) ticarcillin/clavulanate and (**b**) cefotaxime. The cell densities of freshly grown *A. tumefaciens* cells incubated with different concentrations of the antibiotics (250, 500, 750, and 1000 μg mL^{-1}) for 24 h were assayed by measuring the culture absorbance at 600 nm.

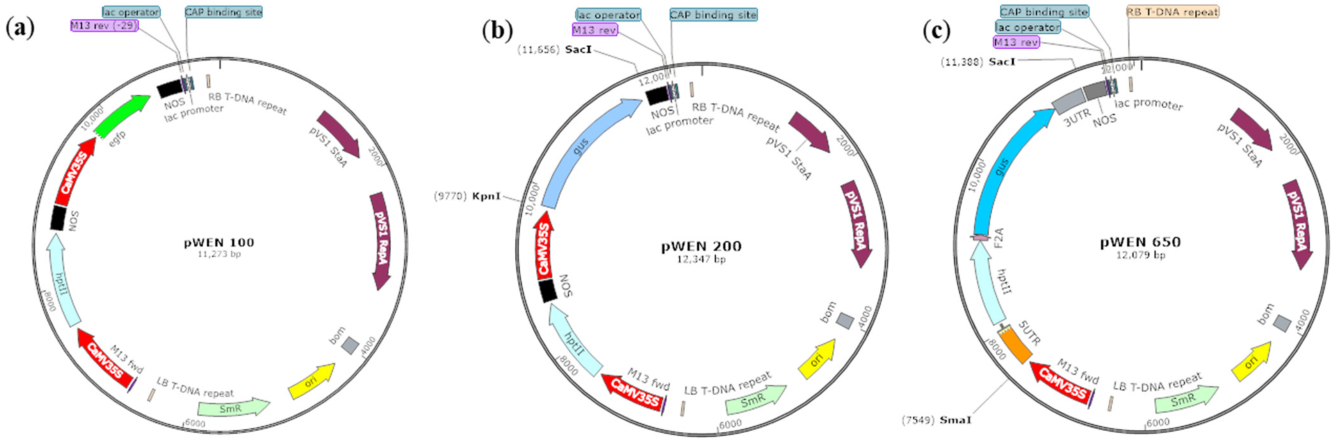

Figure S3. Maps of binary vectors (**a**) pWEN 100, (**b**) pWEN 200, and (**c**) pWEN 650.

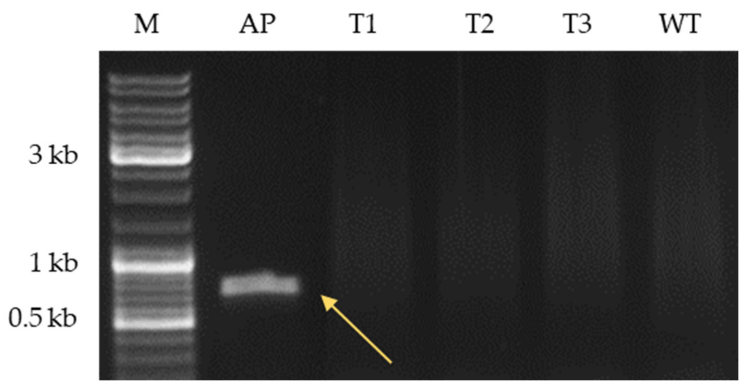

Figure S4. PCR-based confirmation of the absence of *A. tumefaciens* contamination in ticarcillin/clavulanate-treated transformants. PCR analysis was done using *VirC* gene-specific primers. An expected amplicon of 730 bp is observed only in the positive control sample. M - GeneRuler DNA Ladder Mix; AP - positive control (*Agrobacterium* Ti-plasmid); T1-T3 - genomic DNA from transformants; WT - genomic DNA from wild-type.

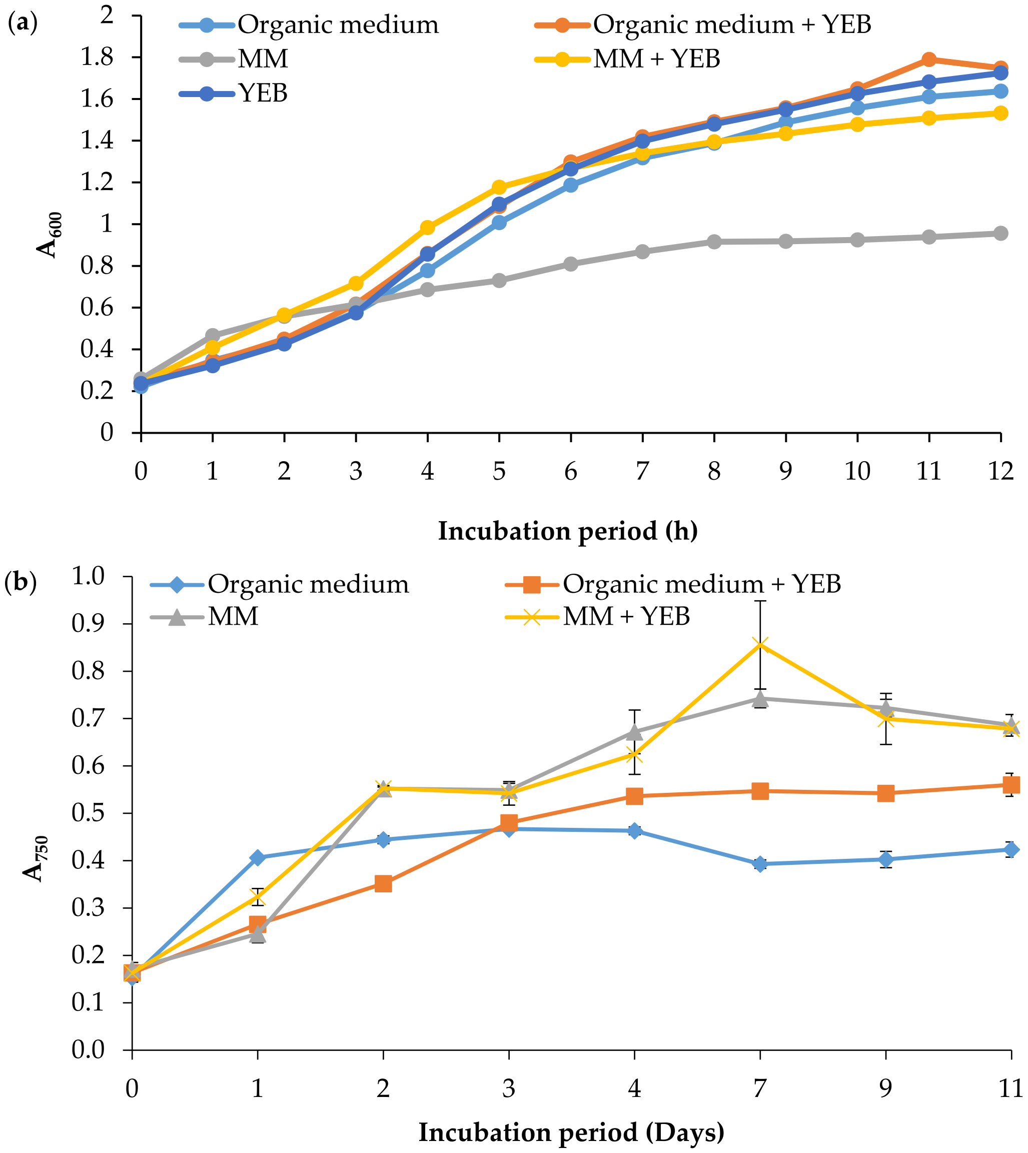

Figure S5. Growth behavior of (**a**) *A. tumefaciens* strain LBA4404 and (**b**) *E. gracilis* in different co-cultivation media (viz. organic medium, MM, organic medium + YEB, MM + YEB). Growth was assayed by measuring the absorbance of the cultures *A. tumefaciens* LBA4404 and *E. gracilis* at 600 nm and 750 nm, respectively, and the mean of three replicates was calculated.

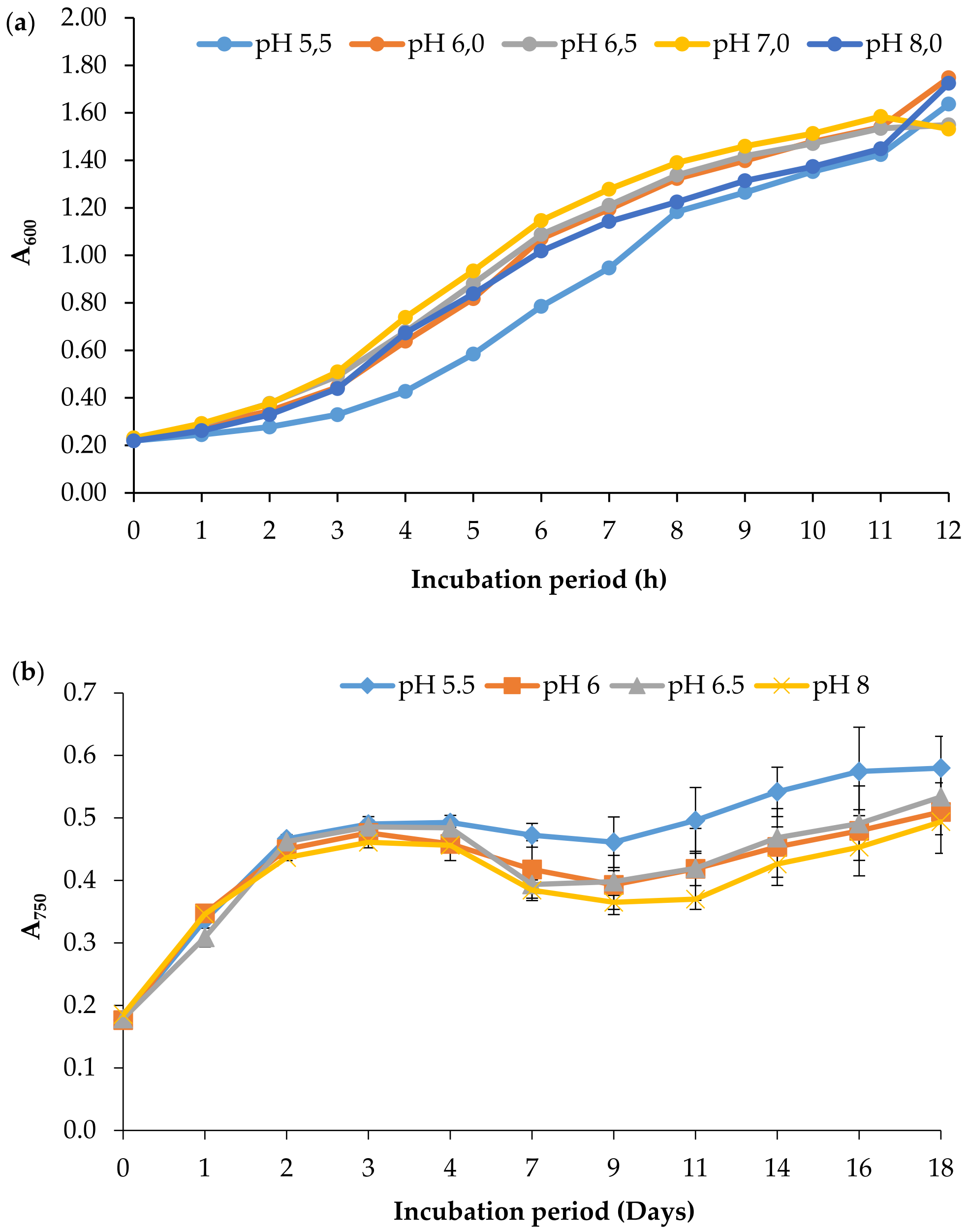

Figure S6. Growth behavior of (**a**) *A. tumefaciens* strain LBA4404 and (**b**) *E. gracilis* in the organic medium of different pH values (5.5, 6.0, 6.5, 7.0, and 8.0). Growth was assayed by measuring the absorbance of the cultures *A. tumefaciens* LBA4404 and *E. gracilis* at 600 nm and 750 nm, respectively, and the mean of three replicates was calculated.

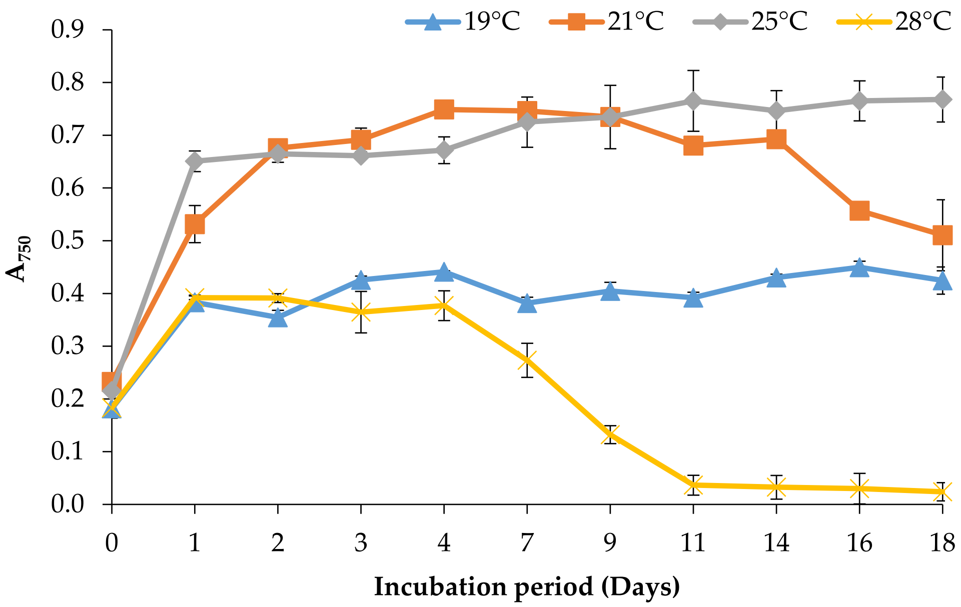

Figure S7. Growth of *E. gracilis* in the organic medium at different temperatures (19, 21, 25, and 28 ºC). Growth was assayed by measuring the culture absorbance at 750 nm, and the mean of three replicates was calculated.

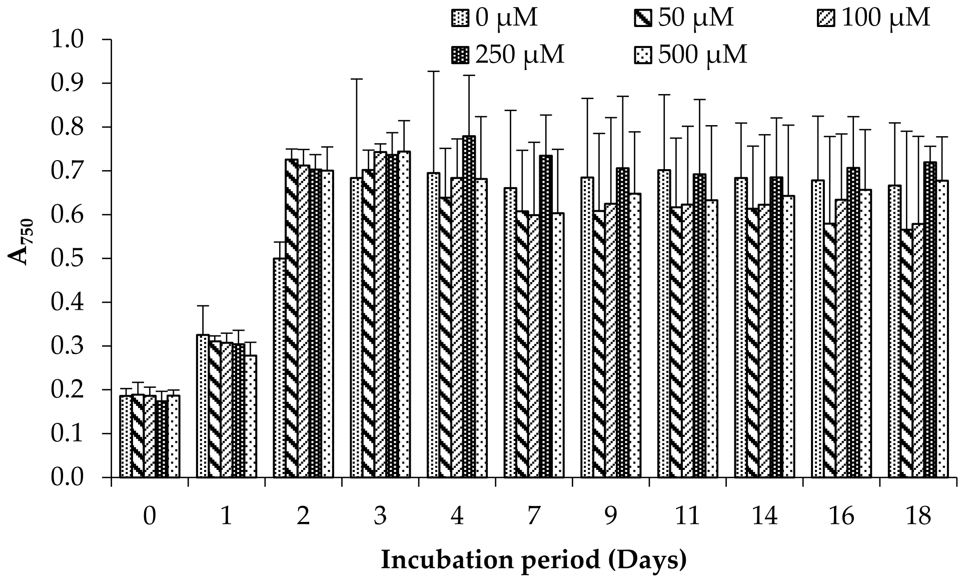

Figure S8. Growth of *E. gracilis* in the organic medium supplemented with various concentrations of acetosyringone. Growth was assayed by measuring the culture absorbance at 750 nm, and the mean of three replicates was calculated.
